# The effects of a myostatin inhibitor on lean body mass, strength, and power in resistance trained males

**DOI:** 10.1186/1550-2783-11-S1-P42

**Published:** 2014-12-01

**Authors:** Matthew Sharp, Ryan P Lowery, Kevin Shields, Jacob Ormes, Sean A McCleary, Jacob Rauch, Jeremy Silva, Ned Arick, Jacob M Wilson

**Affiliations:** 1 The University of Tampa, Tampa, Florida, USA

## Background

Myostatin is considered an inhibitor of satellite cell activation and as a result skeletal muscle hypertrophy. One promising supplement which has suppressed blood levels of myostatin by 44% is a proprietary bioactive ingredient, Myo-T12, which is follistatin derived from fertile chicken egg yolk isolate. MyoT12 would therefore theoretically enhance skeletal muscle growth. However this remains to be examined. Therefore the purpose of this study was to investigate the effects of MyoT12 on skeletal muscle growth and strength in recreationally trained individuals.

## Methods

37 recreationally trained college aged males volunteered to participate in this study and were divided equally into 3 groups receiving a placebo macronutrient matched control, 10 or 30 grams of MYOX (MyoS Corp) supplementation for 8 weeks. All subjects participated in a 12 week periodized resistance training split. DXA determined lean mass, ultrasonography determined muscle mass, and lower and upper body strength were measured pre and post 12 weeks of training. Consent to publish the results was obtained from all participants.

## Results

Results indicated a group X time effect (p<0.05) for lean mass in which the 10 (+1.7 kg) and 30 gram (+1.68 kg), but not placebo (+0.6 kg) groups increased lean mass. Similarly there was a group X time effect for muscle thickness (p<0.05) in which the delta change (post – pre values) increased to a greater extent in the 10 (+ 0.26 cm) and 30 gram (+ 0.2 cm) conditions as compared to the placebo condition (+ 0.06). All groups increased equally in bench press and leg press strength.

## Conclusions

Our findings indicate that MyoX supplementation is efficacious in increasing muscle mass in recreationally trained males. It is likely that the relatively novice subjects in this study experienced the majority of their strength gains via neural adaptations irrespective of changes in muscle size. Future research should investigate the impact of MYOX on females, as well as males who are highly trained and have plateaued in their ability to obtain neural adaptations.

